# Enhanced Polyphenols Recovery from Grape Pomace: A Comparison of Pressurized and Atmospheric Extractions with Deep Eutectic Solvent Aqueous Mixtures

**DOI:** 10.3390/antiox12071446

**Published:** 2023-07-18

**Authors:** Nils Leander Huamán-Castilla, Nicolás Gajardo-Parra, José R. Pérez-Correa, Roberto I. Canales, Maximiliano Martínez-Cifuentes, Gabriela Contreras-Contreras, María Salomé Mariotti-Celis

**Affiliations:** 1 Escuela de Ingeniería Agroindustrial, Universidad Nacional de Moquegua, Prolongación Calle Ancash s/n, Moquegua 18001, Peru; nhuamanc@unam.edu.pe; 2 Chemical and Bioprocess Engineering Department, School of Engineering, Pontificia Universidad Católica de Chile, Vicuña Mackenna 4860, P.O. Box 306, Santiago 7820436, Chile; nfgajardo@ing.puc.cl (N.G.-P.); rocanalesm@ing.puc.cl (R.I.C.); 3 Departamento de Química Orgánica, Facultad de Ciencias Químicas, Universidad de Concepción, Edmundo Larenas 129, Concepción 4070371, Chile; maxmartinez@udec.cl; 4 Escuela de Nutrición y Dietética, Facultad de Medicina, Universidad Finis Terrae, Santiago 7501015, Chile; gcontreras@uft.cl

**Keywords:** grape pomace, polyphenols, antioxidant capacity, deep eutectic solvents, hot pressurized liquid extraction, atmospheric extraction

## Abstract

Deep eutectic solvents (DES) are emerging as potent polyphenol extractors under normal atmospheric conditions. Yet, their effectiveness in hot pressurized liquid extraction (HPLE) must be studied more. We explored the ability of various water/DES and water/hydrogen bond donors (HBDs) mixtures in both atmospheric solid liquid extraction (ASLE) and HPLE (50%, 90 °C) for isolating specific polyphenol families from Carménère grape pomace. We assessed extraction yields based on total polyphenols, antioxidant capacity, and recovery of targeted polyphenols. The HBDs ethylene glycol and glycerol outperformed DES in atmospheric and pressurized extractions. Ethylene glycol exhibited a higher affinity for phenolic acids and flavonols, while flavanols preferred glycerol. Quantum chemical computations indicated that a high-water content in DES mixtures led to the formation of new hydrogen bonds, thereby reducing polyphenol-solvent interactions. HPLE was found to be superior to ASLE across all tested solvents. The elevated pressure in HPLE has caused significant improvement in the recovery of flavanols (17–89%), phenolic acids (17–1000%), and flavonols (81–258%). Scanning electron microscopy analysis of post-extraction residues suggested that high pressures collapse the plant matrix, thus easing polyphenol release.

## 1. Introduction

Chile produces approximately 95 million liters annually of Carménère wine, a beverage widely cherished for its low astringency and deep red hue [1,2]. This production, however, generates around 22 million kilograms of grape pomace—primarily consisting of skins and seeds—which poses significant environmental management challenges as an agro-industrial byproduct [3]. Left unaddressed, these waste materials can lead to various adverse environmental effects, including unpleasant odors, decomposition, soil and water pollution through pollutant leaching, and greenhouse gas emissions [4]. Nevertheless, grape pomace is notably rich in polyphenols, providing an opportunity to extract and incorporate them into nutraceuticals and health-promoting food additives [5,6].

Polyphenols, secondary metabolites with molecular weights ranging from 300 Da to 30,000 Da [7], are abundant in grape pomace and include phenolic acids, flavanols, flavonols, and stilbenes. These compounds are highly sought-after as functional ingredients in the pharmaceutical and food industries due to their capacity to combat oxidative stress-related diseases [8]. For instance, phenolic acids are known to lower the risk of diabetes significantly [9], while flavanols effectively reduce inflammation following infection [9]. Flavonols have been found to help prevent diseases such as osteoporosis and lung cancer [10]. Consequently, developing an environmentally friendly process for extracting polyphenols from plant matrices holds considerable interest for the industry [11].

Polyphenols can be recovered using conventional methods such as atmospheric solid-liquid extraction (ASLE) or greener alternatives like hot pressurized liquid extraction (HPLE). Optimizing key process parameters in HPLE include temperature, pressure, time, and solvent composition [11]. Although both methods may employ the same solvents, their extraction yields can vary significantly. ASLE operates at atmospheric pressure (101.3 kPa) and moderate temperatures (40–80 °C), typically using protic solvents (e.g., ethanol, glycerol, and isopropanol) or their aqueous mixtures to enhance polyphenol extraction [12,13,14]. However, the prolonged processing times of ASLE (over 1 h) could undermine polyphenols recovery due to undesirable oxidation and hydrolysis, thereby diminishing the overall process efficiency [15,16]. In contrast, HPLE emerges as an innovative, environmentally-friendly alternative, operating under subcritical conditions (approximately 10 atm and elevated temperatures between 90–150 °C). This method achieves high extraction yields of polyphenols in notably shorter processing times [11,17,18]. In particular, hydrogen bond donor (HBD) solvents, such as ethanol and glycerol, when applied in HPLE, have demonstrated the ability to recover up to twice the amount of polyphenols compared to ASLE [17,18].

Water-ethanol and water-glycerol mixtures have been extensively studied for their ability to recover significant amounts of polyphenols [19,20]. In this sense, glycerol as an alternative green extraction solvent has been reinforced due to its significantly higher selectivity and yield in the recovery of polyphenols from grape pomace [17]. Glycerol is a byproduct of the fat and oil industry, and its production is cheaper and easier than ethanol. Hence, glycerol is a good option for scaling up the polyphenol extraction process [17]. Nevertheless, the industry constantly seeks new, more sustainable options for extracting these compounds. Deep eutectic solvents (DESs) offer several advantages over traditional solvents, such as ease of preparation and storage, low volatility, biodegradability, and the ability to extract polar and non-polar compounds [21,22]. Furthermore, DES have shown higher recovery yields than organic solvents like glycerol [23].

In the preparation of DES, a hydrogen bond acceptor (HBA) and an HBD are combined, resulting in a strong interaction that produces a liquid from two different solids or a liquid-solid mixture at ambient pressure and room temperature [24]. When primary metabolites, such as sugars and amino acids, are used to form DES, they are called natural deep eutectic solvents (NADES) [25]. In polyphenols recovery using DES, specific HBD:HBA pairings have enhanced extraction yields compared to pure water or organic solvents like ethanol [25,26,27,28]. This increased efficiency is attributed to the formation of hydrogen bonds between the polyphenols and the DES, facilitating intermolecular interactions; adding minimal amounts of DES to water can boost polyphenols’ solubilities [25].

DES and HBD solvents offer significant benefits for polyphenols recovery. However, the costs associated with their preparation can be decisive in choosing between them. For example, producing 1 kg of a mixture of 50% water/DES using a combination of glycerol (22%) and choline chloride (28%) can cost approximately USD 0.039, while preparing 1 kg of a mixture of 50% water/HDB using glycerol (50%) is a bit cheaper (USD 0.015) [29,30]. However, it is also essential to consider the solvents’ ability to interact effectively with polyphenols. Typically, DES are employed under atmospheric conditions to enhance the recovery of total polyphenols. However, the effectiveness and selectivity of DES in conjunction with HPLE for extracting polyphenols from Carménère grape pomace have yet to be investigated. Additionally, the impact of the high pressure of HPLE on the recovery of polyphenols from grape pomace has not been addressed before.

This study evaluates the efficiency of different solvents and extraction methods to recover polyphenols from Carménère grape pomace. Aqueous mixtures of 50 wt.% of various HBDs and DES in ASLE (101.3 kPa) and HPLE (10 MPa) at 90 °C were tested. The extraction yields were determined by measuring the antioxidant capacity, the total polyphenol content, and the recovery of specific low molecular weight polyphenols. Quantum chemical calculations were performed to understand the behavior of DES and their respective HBDs under the evaluated polyphenol extraction conditions. Additionally, surface characterization images were obtained after extraction to elucidate the effects of high pressure and solvents on the grape pomace matrix structure.

## 2. Materials and Methods

### 2.1. Chemicals and Analytic Reagents

High-performance liquid chromatography (HPLC) grade reagents and solvents for extraction and chemical analyses were obtained from Sigma Aldrich (St. Louis, MO, USA), including acetone, acetonitrile, formic acid, acetic acid, methanol, and glycerol. Polyphenol standards, such as catechin (≥98%), epigallocatechin (≥98%), epicatechin (≥98%), kaempferol (≥98%), resveratrol (≥98%), quercetin (≥97%), gallic acid (≥99%), caffeic acid (≥99%), chlorogenic acid (≥98%), vanillic acid (≥99%), protocatechuic acid (≥98%), and ferulic acid (≥98%), were sourced from Xi’an Haoxuan Bio-Tech Co., Ltd. (Shaanxi, China). Choline chloride, levulinic acid, and ethylene glycol, with purities above 99%, were acquired from Acros Organics (Geel, Belgium) for DES preparation. Three DES were prepared using choline chloride as the HBA and levulinic acid, ethylene glycol, and glycerol as the HBDs in a molar ratio of 1:2 of HBA:HBD.

### 2.2. Preparation of Extraction Solvents

Table 1 summarizes the extraction solvents used. DES were prepared gravimetrically using an analytical balance (Practum 224-1s Sartorius, Germany, uncertainty ±0.1 mg). The HBA and HBD were combined under a nitrogen atmosphere and heated at 80 °C until a homogeneous liquid was formed. Choline chloride was dried in a Schlenk line under high vacuum (10^−4^ mbar) to prevent water absorption from ambient humidity before DES preparation. The water content in each DES was measured using a Karl Fischer Coulometer (831KF Metrohm, Herisau, Switzerland). Aqueous mixtures of DES and HBD (50%) were also prepared gravimetrically.

### 2.3. Grape Pomace

Carménère pomace samples were provided by Concha y Toro Vineyard, Region del Maule, Chile, and immediately frozen at 253.15 K after the winemaking process. The samples were then ground to a particle size of less than 1 mm in diameter using a blender (Oster, Sunbeam Products, Inc., Boca Raton, FL, USA).

### 2.4. Atmospheric Solid-Liquid Extraction of Carménère Pomace

A total of 0.5 g (dry weight) of Carménère pomace was extracted at 90 °C for one h in a Carousel 12 Plus™ Reaction Station (Radleys, Saffron Walden, UK) using aqueous mixtures of DES (choline chloride:levulinic acid, choline chloride:ethylene glycol, and choline chloride:glycerol) or their HBD precursors (levulinic acid, ethylene glycol, and glycerol) to achieve a matrix/extractant ratio of 1:10. The extracts were then collected and stored in amber vials at −20 °C.

### 2.5. Hot Pressurized Liquid Extraction of Carménère Pomace

A mixture of 5 g (dry weight) of Carménère pomace and 40 g of quartz sand was placed into a 100 mL cell for extraction using an Accelerated Solvent Extraction device (ASE 150, Dionex, Thermo Fisher Scientific, Waltham, MA, USA) under the following parameters: (i) matrix/solvent ratio of 1:10, (ii) 90 °C, (iii) 250 s of nitrogen purge, (iv) 5 min of static time, (v) one extraction cycle, (vi) 50% of washing volume, and (vii) 10 MPa. Aqueous mixtures of DES and HBD precursors (Table 1) were then injected to extract poly-phenols. The resulting extracts were collected and stored in amber vials at −20 °C.

### 2.6. Total Polyphenol Content (TPC)

The TPC of the extracts was determined using the polyphenol index I_280_. The extracts were diluted with water (0.6:10), and the absorbance was measured directly at 280 nm. A calibration curve was prepared using gallic acid as a standard (10 mg/L–90 mg/L; r2: 0.9793). Results were expressed as mg gallic acid equivalent (GAE) per gram of dried pomace (mg/gdp).

### 2.7. Antioxidant Capacity

The antioxidant capacity of the extracts was determined using spectrophotometry (Spectrometer UV 1240, Shimadzu, Kyoto, Japan) at 517 nm with the DPPH radical scavenging method [31]. In summary, 0.1 mL of diluted extract and 3.9 mL of DPPH solution (0.1 mM) were mixed, and then the mixture was protected from light for 30 min at room temperature. Two controls were used for this assay; (i) 3.9 mL of methanol (dilution blank) and (ii) 3.9 mL of the methanolic solution of DPPH (negative control). The effective extract concentration required to inhibit 50% of the DPPH radical absorption (IC50; mg/L) was calculated. The extract antioxidant capacity was compared with Trolox using the Trolox equivalent antioxidant capacity (TEAC) equation: TEAC = IC50 Trolox/IC50 sample. DPPH values were expressed as µmol of Trolox equivalent (TE) per gram of dry mass of pomace (µM TE/gdp).

### 2.8. Target Polyphenols Quantification

The content of gallic acid, catechin, epigallocatechin, epicatechin, kaempferol, resveratrol, quercetin, caffeic acid, and chlorogenic acid in ASLE and HPLE extracts was quantified using Ultra Performance Liquid Chromatography coupled with a Mass detector (UPLC-MS) [32]. A 1 mL aliquot of the extracts was diluted with distilled water (1:10) and filtered through a 0.22 µm membrane. A 5 µL sample of the filtered solution was then injected into a UHPLC-MS system (Dionex Ultimate 3000 with Detector MS Orbitrap Exactive Plus, Thermo Fisher, Bremen, Germany) equipped with a reverse-phase Acquity UHPLC BEH C18 column (1.7 µm × 2.1 × 100 mm) maintained at 308.15 K. Chromatographic separation conditions involved a mobile phase comprising A (acetonitrile and 0.1% formic acid) and B (water and 0.1% formic acid) in a gradient elution analysis programmed as follows: 80% A–20% B for 6 min, 15% A–85% B for 18 min, and 80% A–20% B maintained for 30 min, at a flow rate of 0.2 mL/min. Calibration curves were obtained by plotting peak areas against nine different concentrations of standard solutions. Analy-ses were performed in triplicate, and results were expressed in µg of specific polyphenol per gram of dry pomace.

### 2.9. Scanning Electron Microscope (SEM) Analysis

The morphology of the post-extraction pomace surfaces was investigated using an FEI Quanta FEG 250 (FEI Corporate, Hillsboro, OR, USA) scanning electron microscope with a maximum resolution of 1 nm. Post-extraction dry grape pomace experiments were conducted for ASLE and HPLE using aqueous mixtures of HBD2 as the solvent. The samples did not require prior treatment to increase their conductivity.

### 2.10. Statistical Analysis

A completely randomized design was employed to determine the effect of extraction solvents (aqueous DES and aqueous HBD precursors) on the total polyphenols content, DPPH, and specific polyphenols. Mean and standard deviation (SD) results were presented. Analysis of variance (ANOVA) and least significant difference tests were applied to the response variables (*p* < 0.05). Statistical data analyses were performed using Statgraphics Plus for Windows 4.0 (Statpoint Technologies, Inc., Warrenton, VA, USA).

### 2.11. Quantum Chemical Calculations

Density functional theory (DFT) calculations were employed to obtain binding energies of the complex between choline chloride:levulinic acid (DES1), choline chloride:ethylene glycol (DES2), choline chloride:glycerol (DES3), and choline chloride with waters molecules. Geometrical optimization was carried out using the Gaussian 09 software (revision a.01; Gaussian, Inc.: Wallingford, CT, USA) [33] at DFT M06-2X/6-311G(d,p) level of theory. No imaginary vibrational frequencies were found at the optimized geometries, indicating that they are the true minimal of the potential energy surface. The binding energies were calculated using the following equation:

E_bind_ (kcal/mol) = E_ab_ − E_a_ − E_b_ + E_BSSE_

where E_bind_ corresponds to the binding energy of the complex, E_ab_ is the total energy of the complex, E_a_ is the energy of choline chloride, E_b_ is the energy of HBD (levulinic acid, ethylene glycol or glycerol), and E_BSSE_ corresponds to the Basis Set Superposition Error (BSSE) correction energy calculated with the counterpoise method [34,35].

## 3. Results

### 3.1. Effect of Aqueous DES and Aqueous HBD Precursors on the Extraction of TPC under ASLE and HPLE Conditions

Using aqueous mixtures of DES and HBD precursors has been shown effective in extracting polyphenols from plant sources. In this study, the utilization of aqueous mixtures of DES and their corresponding HBDs during the ASLE of Carménère pomace did not exhibit a clear trend in the extraction of TPC (Table 2). DES3 (choline chloride + glycerol) yielded a TPC recovery rate that was approximately 28% and 19% higher than that of DES1 (choline chloride + levulinic acid) and DES2 (choline chloride + ethylene glycol), respectively (Table 2). However, using HBD3 (glycerol) significantly increased the total polyphenol recovery rate by approximately 8% compared to DES3 (Table 2). The chloride anions in the choline chloride structure likely have a greater preference for interacting with the hydroxyl groups of glycerol, which could affect the ability of DES to interact with polyphenols [36]. Since Florindo et al. [37] and Jessop et al. [38] reported that solvent polarity parameters for DES3 (choline chloride + glycerol) and HBD3 (glycerol) expressed as π* (polarizability), α (acidity), and β (basicity) are similar, it is necessary to consider other physical properties associated with molecular interactions between polyphenols and solvents to explain this behavior.

Under HPLE conditions, the assessed hydrogen bond donor (HBD) precursors demonstrated greater efficiency in extracting polyphenols than their respective DES counterparts (refer to Table 2). HPLE extracts obtained with HBD2 and HBD3 exhibited the highest TPC values, which were not significantly different (*p* > 0.05). Specifically, using HBD2 or HBD3 precursors resulted in extracts with higher TPC yields, corresponding to approximately 4%, 15%, and 17% higher TPC content compared to DES3, DES2, and DES1, respectively (refer to Table 2). Pal et al. [39] observed that choline chloride and glycerol interactions decrease as the temperature increases from 40 to 120 °C. It could be hypothesized that under subcritical conditions, hydrogen bond interactions between the HBD (glycerol) and HBA (choline chloride) are weakened, thereby affecting the density, polarity, and solubility of DES and, consequently, its ability to solubilize polyphenols. In this context, Ozturk et al. [40] observed that an increase in water content (from 30% to 50%) of DES (glycerol + choline chloride) led to a 40% decrease in its ability to recover polyphenols. Gajardo et al. [41] reported that when water content exceeds 30% by weight, a new network of hydrogen bonds forms between the water molecules and the mixture components. This new network can disrupt the existing network of choline chloride and glycerol, which can reduce the solubility properties of the mixture, recovering less polyphenols.

To theoretically corroborate the effect of water content on DES selectivity for polyphenols, we quantitatively compared choline chloride preferences for water and the HBD in the respective DES. Quantum chemical calculations were performed to determine the binding energies of the following complexes: choline chloride:levulinic acid, choline chloride:ethylene glycol, and choline chloride:glycerol. Additionally, we calculated the binding energy of choline chloride solvated by water molecules. Three water molecules were considered explicitly to simulate the solvation of choline chloride at a reasonable computational cost. The binding energies were calculated using DFT M06-2x/6-311G(d,p) level of theory and are presented in Table 3. Our findings reveal that the strongest interaction among these DESs corresponds to number 2 (choline chloride:ethylene glycol), showing approximately half the binding energy observed for choline chloride:water. This is consistent with our experiments since a relatively high percentage of water (50% *w*/*w*) was employed in the mixture with the DES. This high-water content likely resulted in the complete disruption of the microstructure and nanostructure of the DES, thus explaining the low recovery achieved with them [42].

### 3.2. Effect of Aqueous DES and Aqueous HBD Precursors on the Antioxidant Capacity of Carménère Pomace Extracts Obtained under ASLE and HPLE Conditions

Some studies have suggested a direct relationship between the antioxidant capacity and the total polyphenol content of natural extracts [43,44,45]. The DPPH method measures the polyphenols’ capacity to scavenge and neutralize DPPH radicals [46,47,48]. In this study, the DPPH values exhibited a behavior similar to that of TPC.

The antioxidant capacity of ASLE extracts obtained with aqueous DES was significantly lower than that of their HBD precursor counterparts. HBD2 (ethylene glycol) and HBD3 (glycerol) extracts showed the highest antiradical activity against DPPH when extraction was carried out at atmospheric pressure (Table 4). Although there were no significant differences (*p* > 0.05) in the antioxidant capacities of these extracts, they were significantly higher than their corresponding DES (Table 4). The high pressure of HPLE increased the antioxidant capacity of the extracts, reaching maximum values of 139.02 and 140.39 µM TE/gdp for the HBD2 and HBD3 extracts, respectively (Table 4). These results outperform those obtained with aqueous mixtures with low ethanol concentrations (23 mg GAE/gdp) [18]. Moreover, the antioxidant capacity obtained in the extracts was of the same order of magnitude as those obtained with water at 423.15 K (130 µM TE/gdp) by Vergara et al. [43]. Previous studies have suggested that HPLE reduces solvent polarity, promoting favorable interactions between the hydroxyl groups of the solvents and some functional groups in polyphenols. In addition, the high pressure applied in the HPLE process increases the kinetic energy of the molecules, leading to enhanced solubility [49,50].

**Figure 1:**
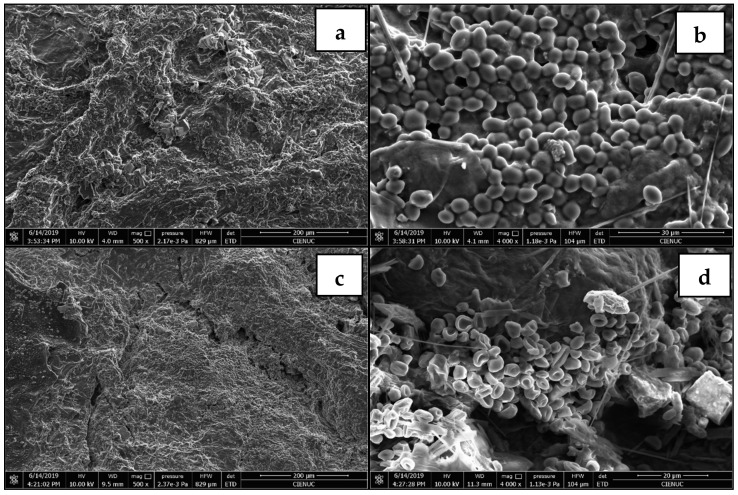
(**a**,**b**) Scanning electron microscope images of post-atmospheric extraction. The scales were established at 200 µm and 30 µm. (**c**,**d**) Post-HPLE process using HBD 2 as solvent. The scales were established at 200 µm and 20 µm.

To better understand the effect of pressure on the extraction yield of polyphenols and the antioxidant capacity of extracts, we carried out an image analysis using scanning electron microscopy (SEM) to examine the grape pomace cake obtained from both extraction methods. The SEM images confirmed that, unlike ASLE, HPLE facilitated the rupture of cellular vacuoles, which likely resulted in a greater release of phenolic compounds and, therefore, a higher antioxidant capacity (Figure 1). Our findings emphasize the importance of considering both solvent composition and the structure of the plant matrix when using HPLE to extract polyphenols [51].

### 3.3. Effect of Aqueous DES and Aqueous HBD Precursors Target Polyphenols of Carménère Pomace Extracts Obtained under ASLE and HPLE Conditions

Carménère pomace extracts contain various phenolic acids, flavanols, and flavonols with biological and technological properties [17]. These specific target polyphenols were found to withstand the applied extraction temperatures in ASLE [52] and HPLE [20] without experiencing any degradation. Based on this observation, the present study analyzed the effect of aqueous DES and aqueous HBD precursor counterparts on specific polyphenols from these families under ASLE and HPLE conditions (Table 5).

#### 3.3.1. Phenolic Acids

This study found that phenolic acids were the least extracted family of polyphenols across all solvents. Due to their low polarity, phenolic acids require solvents with lower polarizability to be effectively extracted. However, this study employed solvents with higher polarizability than those used in previous research, leading to lower extraction yields for phenolic acids [18,50].

Regarding absolute concentrations, HBDs precursors extracted significantly more phenolic acids than their corresponding DES in ASLE and HPLE conditions (Table 5). For example, in ASLE, using HBD1, HBD2, and HBD3 precursors allowed for the extraction of 66, 9.8, and 6.5 times more phenolic acids than their respective DES. This difference was maintained under HPLE conditions, but to a lesser extent, with an extraction 5.9, 9.4, and 6.7 times greater when extracting with HBD1, HBD2, and HBD3, respectively, compared to their corresponding DES.

When comparing the effect of pressure on the absolute concentration of phenolic acids, HPLE significantly favored their recovery from Carménère pomace. HBD2 and HBD3 in HPLE presented the highest values of extracted phenolic acids (Table 5). Interestingly, HBD2 extracts showed about 25 times more gallic acid than other phenolic acids. The polar nature, low viscosity, and presence of two hydroxyl groups in HBD2 likely enhance its ability to interact with the functional groups of specific polyphenol families, such as phenolic acids and flavonols [40].

#### 3.3.2. Flavanols

Flavanols were the most extracted polyphenol family, independent of the solvent and extraction pressure applied (Table 5). While grape pomace contains substantial amounts of phenolic acids and flavonols, these are primarily present in the form of gallated procyanidins, which makes them available as flavanols [50]. HBDs extracted a significantly greater amount of flavanols compared to their corresponding DESs in both ASLE and HPLE. For example, HBD1, HBD2, and HBD3 extracted 63, 94, and 62 times more flavanols than DES1, DES2, and DES3 in ASLE, respectively (Table 5); similarly, in HPLE, HBD1, HBD2, and HBD3 extracted 85, 86, and 65 times more flavanols than DES1, DES2, and DES3, respectively. The pressurized system obtained higher yields than the atmospheric process, regardless of the solvent used. When comparing HPLE vs. ASLE using DES1, DES2, and DES3, 17%, 56%, and 84% more flavanols were obtained, respectively. The impact of pressure was more significant with HBD solvents, increasing flavanol extraction by 62%, 43%, and 88% with HBD1, HBD2, and HBD3, respectively. HBD2 and HBD3 in HPLE conditions produced extracts with the highest concentrations of flavanols, 33.84 µg/gdp and 38.56 µg/gdp, respectively.

#### 3.3.3. Flavonols

HBDs extracted significantly higher levels of flavonols than their respective DESs under ASLE and HPLE conditions (Table 5). HBD2 and HBD3 ASLE extracts contained 23- and 11-fold more flavonols than DES2 and DES3 ASLE extracts. HBD2 and HBD3 HPLE extracts contained 2- and 1.5-fold more flavonols than DES2 and DES3 HPLE extracts. HBD2 extracts contained the highest amounts of flavonols (10.43 µg/gdp in ASLE, 18.88 µg/gdp in HPLE). DES did not achieve high yields for the target polyphenol families, except for quercetin and kaempferol. This may be because some DES are not as efficient as their HBD (hydrogen bond donor) precursor solvents in forming strong hydrogen bonds with polyphenols, which can lead to lower extraction yields. Furthermore, the formation of hydrogen bonds between DES components can compete with the formation of hydrogen bonds with the polyphenols, further reducing extraction efficiency. Nevertheless, the application of high pressures increased more than three times the extraction capacity of the DES. The complexity of the extraction matrix, which contains various high molecular-weight polyphenolic compounds, may also play a role in the observed behavior. While we obtained high TPC and AC values, the UPLC-MS analysis could not quantify all the polyphenolic compounds in the extraction matrix.

## 4. Conclusions

This study compared the extraction performances between DES and the corresponding HBD solvents using ASLE and HPLE systems. Ethylene glycol and glycerol, as HBD solvents, exhibited superior extraction yields across all experimental conditions. More specifically, ethylene glycol excelled in recovering phenolic acids and flavonols, whereas glycerol demonstrated selective extraction of flavanols. Quantum chemical calculations revealed that an increased water proportion in DES mixtures favored new hydrogen bond formations, consequently diminishing polyphenol-solvent interactions. HPLE consistently outperformed ASLE in terms of extraction effectiveness for all tested solvents. HPLE-acquired extracts presented higher concentrations of flavanols (17–89%), phenolic acids (17–1000%), and flavonols (81–258%), as well as enhanced antioxidant capacity (AC) (34–79%) and total polyphenol content (TPC) (21–35%). Notably, no flavonols were found in DES1/ASLE extracts, in contrast to the DES1/HPLE extract, which contained 5.96 g/gdp of flavonols. The Scanning Electron Microscopy (SEM) analysis of post-extraction residues indicated that high-pressure conditions induced a collapse in the plant matrix, thus promoting polyphenol release. These observations offer crucial insights for optimizing and scaling up the polyphenol extraction process, ultimately aiding in developing efficient and sustainable extraction methodologies.

## Figures and Tables

**Table 1:** Composition of the solvent mixtures used in the extractions.

Extraction Solvent	Co-Solvent Description		Mass Fraction		
	HBA	HBD	Water	HBA	HBD
DES1	Choline chloride	Levulinic Acid	0.5	0.19	0.31
DES2	Choline chloride	Ethylene glycol	0.5	0.25	0.24
DES3	Choline chloride	Glycerol	0.5	0.22	0.28
HBD1	-	Levulinic Acid	0.5	-	0.5
HBD2	-	Ethylene glycol	0.5	-	0.5
HBD3	-	Glycerol	0.5	-	0.5

HBA: hydrogen bond acceptor. HBD: hydrogen bond donor. DES: Deep eutectic solvent.

**Table 2:** Total polyphenol content of ASLE and HPLE extracts.

Description	TPC (mg GAE/gdp)	
	ASLE	HPLE
DES1	33.39 ^a^ ± 0.59	51.62 ^a^ ± 1.53
DES2	37.62 ^a,b^ ± 1.03	55.60 ^b^ ± 0.89
DES3	46.39 ^c^ ± 0.79	56.41 ^b^ ± 1.33
HBD1	35.63 ^a^ ± 2.02	52.80 ^a^ ± 2.13
HBD2	46.11 ^c^ ± 1.55	62.44 ^c^ ± 1.67
HBD3	49.22 ^c^ ± 1.13	62.38 ^c^ ± 2.13

ASLE: Atmospheric Solid-Liquid Extraction. HPLE: Hot Pressurized Liquid Extraction. DES: Deep eutectic solvent. HBD: hydrogen bond donor. TPC: Total polyphenols content was expressed as mg of gallic acid equivalent (GAE) per gram of dry pomace (gdp). The results are expressed as the mean and SD (standard deviation). Different letters indicate statistically significant differences between columns (*p* < 0.05).

**Table 3:** Binding energies for the complexes, calculated at DFT M06-2x/6-311G(d,p) level.

Complex	Binding Energy (kcal/mol)
DES1	−15.4
DES2	−19.1
DES3	−17.1
Choline chloride:water	−37.4

**Table 4:** Antioxidant capacity (DPPH) of ASLE and HPLE extracts.

Description	DPPH µM TE/gdp	
	ASLE	HPLE
DES1	18.32 ^a^ ± 1.63	93.17 ^a^ ± 2.54
DES2	26.90 ^b^ ± 3.18	118.18 ^c^ ± 3.84
DES3	24.96 ^b^ ± 2.01	121.73 ^c^ ± 2.89
HBD1	31.12 ^c^ ± 2.63	112.24 ^b^ ± 3.11
HBD2	56.12 ^d^ ± 1.88	139.02 ^c,d^ ± 4.85
HBD3	57.48 ^d^ ± 2.05	140.39 ^d^ ± 3.72

DPPH was expressed as micromole of Trolox equivalent per gram of dry weight. The results are expressed as the mean and SD (standard deviation). Different letters indicate statistically significant differences between columns (*p* < 0.05).

**Table 5:** Polyphenol’s profile of ASLE and HPLE extracts obtained using aqueous DES and HBD mixtures.

Description	DES 1		DES 2		DES 3		HBD 1		HBD 2		HBD 3	
	ASLE	HPLE	ASLE	HPLE	ASLE	HPLE	ASLE	HPLE	ASLE	HPLE	ASLE	HPLE
**Phenolic acids (µg/gdp)**	Mean ± SD	Mean ± SD	Mean ± SD	Mean ± SD	Mean ± SD	Mean ± SD	Mean ± SD	Mean ± SD	Mean ± SD	Mean ± SD	Mean ± SD	Mean ± SD
Gallic	0.01 ± 0.01	0.17 ± 0.01	0.14 ± 0.01	0.24 ± 0.01	0.17 ± 0.01	0.26 ± 0.02	1.26 ± 0.05	1.45 ± 0.06	1.40 ± 0.05	2.56 ± 0.14	1.17 ± 0.02	2.44c ± 0.33
Caffeic	0.01 ± 0.01	0.05 ± 0.00	0.01 ± 0.00	0.05 ± 0.00	0.02 ± 0.01	0.06 ± 0.01	0.05 ± 0.01	0.08 ± 0.01	0.07 ± 0.01	0.10 ± 0.02	0.05 ± 0.01	0.07 ± 0.01
Chlorogenic	nd	nd	nd		nd	0.04 ± 0.01	nd	0.03 ± 0.01	nd	0.08 ± 0.01	nd	0.04 ± 0.01
Ʃ:	**0.02** **± 0.01**	**0.22** **± 0.01**	**0.15** **± 0.02**	**0.29** **± 0.01**	**0.19** **± 0.01**	**0.38** **± 0.02**	**1.31** **± 0.04**	**1.53** **± 0.05**	**1.47** **± 0.03**	**2.74** **± 0.08**	**1.22** **± 0.02**	**2.55** **± 0.16**
**Flavanols (µg/gdp)**												
Epigallocatechin	0.12 ± 0.01	0.15 ± 0.04	0.10 ± 0.01	0.18 ± 0.01	0.13 ± 0.00	0.24 ± 0.05	7.92 ± 0.74	16.28 ± 1.29	11.33 ± 0.96	16.83 ± 1.58	11.95 ± 0.62	22.43 ± 2.39
Catechin	0.05 ± 0.01	0.08 ± 0.02	0.05 ± 0.01	0.09 ± 0.01	0.06 ± 0.02	0.17 ± 0.01	3.65 ± 0.11	5.76 ± 0.21	4.23 ± 0.13	6.23 ± 0.39	2.67 ± 0.19	6.01 ± 0.37
Epicatechin	0.12 ± 0.01	0.11 ± 0.01	0.10 ± 0.01	0.12 ± 0.02	0.13 ± 0.01	0.18 ± 0.01	6.74 ± 0.02	7.68 ± 0.42	8.04 ± 0.51	10.78 ± 1.00	5.82 ± 0.28	10.12 ± 0.64
Ʃ:	**0.29** **± 0.01**	**0.34** **± 0.03**	**0.25** **± 0.01**	**0.39** **± 0.01**	**0.32** **± 0.01**	**0.59** **± 0.03**	**18.31** **± 0.44**	**29.72** **± 0.98**	**23.60 ** **± 0.42**	**33.84** **± 0.78**	**20.44** **± 0.29**	**38.56** **± 0.21**
**Flavonols (µg/gdp)**												
Quercetin	nd	5.49 ± 1.35	nd	nd	2.23 ± 0.48	1.42 ± 0.19	5.30 ± 1.22	10.91 ± 0.34	9.46 ± 0.83	13.69 ± 0.91	3.95 ± 0.06	8.98 ± 0.78
Kaempferol	nd	0.47 ± 0.07	0.45 ± 0.08	1.61 ± 0.11	nd	5.39 ± 0.60	0.61 ± 0.06	1.49 ± 0.05	0.97 ± 0.18	5.19 ± 0.32	0.40 ± 0.02	1.31 ± 0.10
Ʃ:		**5.96** **± 0.72**	**0.45** **± 0.08**	**1.61** **± 0.11**	**2.23** **± 0.48**	**6.81** **± 0.43**	**5.91** **± 0.83**	**11.40** **± 0.17**	**10.43** **± 0.39**	**18.88** **± 0.56**	**4.35** **± 0.03**	**10.29** **± 0.43**

PC: Specific polyphenols Content was expressed as µg per gram of dry pomace. HPLE: hot pressurized liquid extraction. ASLE: Atmospheric solid-liquid extraction, SD: standard deviation (*n* = 3), nd: non detected.

## Data Availability

The data presented in this study are available in the article.
